# IRAK-4 inhibition: emavusertib for the treatment of lymphoid and myeloid malignancies

**DOI:** 10.3389/fimmu.2023.1239082

**Published:** 2023-10-26

**Authors:** Ricardo D. Parrondo, Madiha Iqbal, Reinhard Von Roemeling, Christina Von Roemeling, Han W. Tun

**Affiliations:** ^1^ Department of Hematology-Oncology, Mayo Clinic Cancer Center, Jacksonville, FL, United States; ^2^ Curis Inc, Lexington, MA, United States; ^3^ Department of Neurosurgery, University of Florida, Gainesville, FL, United States

**Keywords:** non-Hodgkin lymphoma, myeloid malignancies, TLR signaling, IRAK 4, small molecule inhibitors

## Abstract

Several studies have identified mutations in the MYD88L265P gene as a key driver mutation in several B-cell lymphomas. B-cell lymphomas that harbor the MYD88L265P mutation form a complex with phosphorylated Bruton’s tyrosine kinase (BTK) and are responsive to BTK inhibition. However, BTK inhibition in B-cell lymphomas rarely results in a complete response and most patients experience eventual disease relapse. Persistent survival signaling though downstream molecules such as interleukin 1 receptor-associated kinase 4 (IRAK-4), an integral part of the “myddosome” complex, has been shown to be constitutively active in B-cell lymphoma patients treated with BTK inhibitors. Emerging evidence is demonstrating the therapeutic benefit of IRAK-4 inhibition in B-cell lymphomas, along with possibly reversing BTK inhibitor resistance. While MYD88 gene mutations are not present in myeloid malignancies, downstream overexpression of the oncogenic long form of IRAK-4 has been found in acute myeloid leukemia (AML) and myelodysplastic syndromes (MDS), particularly in AML and MDS that harbor mutations in splicing factors U2AF1 and SF3B1. These data suggest that the anti-leukemic activity of IRAK-4 inhibition can be exploited in relapsed/refractory (R/R) AML/MDS. In this review article, we discuss the currently available pre-clinical and clinical data of emavusertib, a selective, orally bioavailable IRAK-4 inhibitor in the treatment of R/R B-cell lymphomas and myeloid malignancies.

## 1 Introduction

Considerable advances made in the understanding of the biological heterogeneity of B-cell non-Hodgkin lymphomas (NHL) and myeloid malignancies such as AML and MDS have translated into a multitude of novel, targeted therapeutic approaches over the past two decades. Despite these efforts, many B-cell NHL and most myeloid malignancies remain incurable with the available therapeutic approaches and patients afflicted with these hematologic malignancies succumb to relapsed/refractory disease. Continued investigation into the mechanisms of lymphomagenesis and leukemogenesis as well as mechanisms of resistance to therapeutics are warranted for these disorders. Abnormal innate immune activation and proinflammatory signaling within the malignant clone have been identified as key oncogenic drivers in B-cell NHL and in myeloid neoplasms (1, 2). Toll-like receptors (TLRs) are a family of pattern recognition receptors that play a key role in the innate immune system (3). Augmented or aberrant TLR signaling is associated with ineffective hematopoiesis and development of hematopoietic malignancies by promoting pro-tumoral inflammation in the tumor microenvironment as well as cell proliferation and survival via downstream activation of the mitogen-activated protein kinase (MAPK) pathway and the canonical NF-kB pathway (4).

IRAK-4 is a key mediator of TLR signaling processes. Following the engagement of TLR agonists, IRAK4 is recruited to the adaptor protein MYD88 through death-domain interactions and IRAK-4, together with other adaptor proteins such as IRAK2, and MYD88, form a signaling complex, termed the “myddosome.” Many B-cell NHL and myeloid malignancies with spliceosome mutations have augmented myddosomal signaling of which IRAK-4 plays a key role (5, 6). Due to the inability of current therapeutics to cure the majority of B-cell NHLs and myeloid neoplasms and the eventual development of drug resistance, the therapeutic targeting of IRAK-4 is a merited treatment strategy.

Emavusertib (CA-4948) is a selective small molecule inhibitor of IRAK-4 that is orally bioavailable. Several phase I/II clinical trials evaluating the safety, efficacy, and pharmacokinetics of emavusertib monotherapy as well as emavusertib in combination with other agents in relapsed/refractory B-cell NHLs and myeloid malignancies are underway. In this review article, we review TLR signaling and summarize all the available pre-clinical and clinical data evaluating the use of emavusertib in hematologic malignancies.

## 2 TLR and BCR signaling in B-cell non-Hodgkin lymphomas

**Figure 1:**
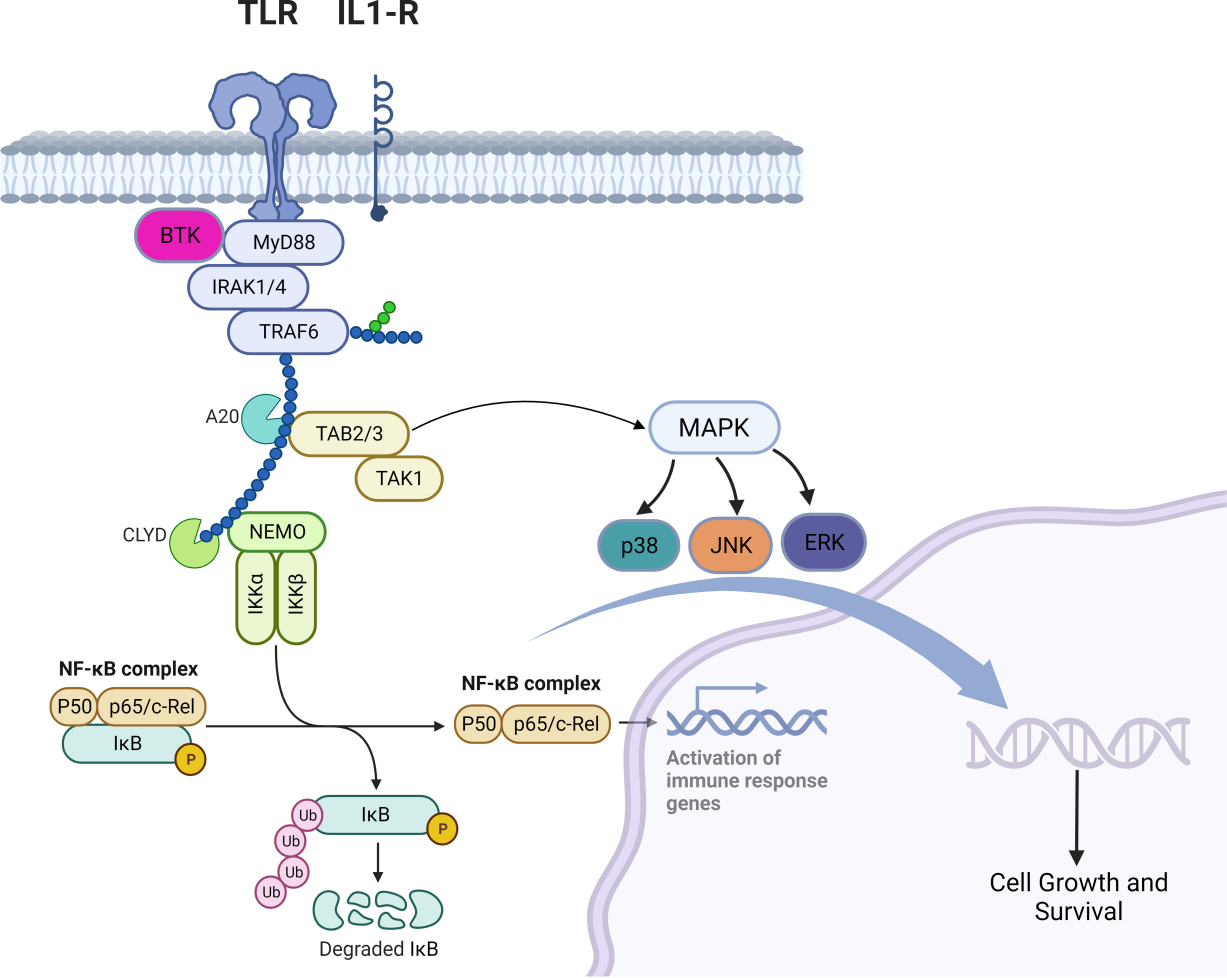
Myddosomal signaling in B-cell malignancies.

TLRs are a family of receptors that play a crucial role in a hosts’ defense against foreign pathogens. TLRs share common structural domains such as an amino (N)-terminal extracellular domain which facilitates ligand recognition, a single transmembrane (TM) domain, and a carboxyl (C)-terminal intracellular Toll/interleukin-1 (IL-1) receptor (TIR) domain that plays a role as a scaffold for the recruitment of downstream signaling proteins (7). TLRs’ downstream signaling is aided by conformational changes in their intracellular TIR domains (changes induced by ligand binding), which ultimately recruits adaptor proteins (8). Myeloid differentiation primary response protein 88 (MYD88) is a central cytoplasmic signaling adaptor for both (TLRs) and (IL1Rs) family proteins (9, 10). When a ligand binds to a TLR, the cytoplasmic TIR domain of the TLRs or IL1R associates with the TIR of MYD88 (11) (12). Subsequently, IL1R-associated kinase 4 (IRAK-4), is recruited to MYD88 and in turn, IRAK-4 phosphorylates IRAK-2 and IRAK-1 to form a structure known the “myddosome” (13). Phosphorylated IRAK-1 and IRAK-2 bind with the E3 ubiquitin ligase TNF receptor–associated factor 6 (TRAF6) and TRAF6 attracts TAK1 binding protein 2 (TAB2) which in turn activates TAB2- associated TGFβ-activated kinase 1 (TAK1) which ultimately promote cellular proliferation and survival via activation of the MAPK pathway and the canonical NF-kB pathway (14–18) (**Figure 1**). B-cell receptor (BCR) signaling and TLR signaling converge in some B-cell non-Hodgkin lymphomas (NHL) and signaling via both pathways are key players in driving malignant B-cell growth. The BCR is essentially a membrane‐anchored antibody that can transduce intracellular signals via non‐covalent interactions with the CD79A‐CD79B heterodimer (5). Recurrent mutations affecting the ITAM motifs of CD79B and CD79A (frequently seen in the activated B cell subtype [ABC subtype] of diffuse large B cell lymphoma) potentiate BCR signaling (19, 20). This “active BCR signalosome” recruits a multitude of adapter/scaffold proteins as well as additional kinases, including phosphatidylinositol 3‐kinase (PI3K), AKT, and Bruton’s tyrosine kinase (BTK) (21). Calcium signaling and subsequent NF‐κB activation is the main downstream pathway of BCR activation (21). BTK signaling plays a crucial role in both BCR and TLR signaling. In some B-cell lymphomas, chronic BCR signaling and constitutive MYD88/TLR signaling come together on IKK (**Figure 1**) culminating in NF‐κB activation (5).

An activating missense mutation (L265P) changing leucine to proline at position 265 in MYD88 has been identified as a driver mutation that promotes B-cell growth and survival in several B-cell NHLs including the ABC subtype of diffuse large B cell lymphoma (DLBCL), Waldenström’s macroglobulinemia/lymphoplasmacytic lymphoma, primary central nervous system lymphoma (PCNSL), cutaneous DLBCL leg type, and testicular DLBCL (22–26). The MYD88L265P mutation is also found in MALT lymphomas and chronic lymphocytic leukemia (CLL) but to a much lesser extent (27–29). Mutations in MYD88L265P are not found in mantle cell lymphomas (30). In these B-cell NHLs with MYD88L265P mutation and even those without, BTK inhibitors such as ibrutinib, acalabrutinib, and zanubrutinib have shown efficacy by virtue of inhibiting BTK’s key role in in BCR and TLR signaling (31–37). However, BTK inhibition does not lead to deep responses and despite their substantial efficacy, most patients eventually develop progressive disease due to primary or acquired resistance (38). Furthermore, constitutive TLR signaling even in the face of BTK inhibition may explain the low response rates in some lymphomas (39). Alternate mechanisms of inhibiting TLR signaling are warranted in B-cell NHL.

### 2.1 Diffuse large B cell lymphoma activated B cell subtype

ABC-DLBCL (29% have MYD88L265P mutations) and extranodal large cell lymphomas (which have an ABC-like gene expression profile) are highly enriched for MYD88L265P, including primary central nervous system lymphoma (PCSNL; ~56%), primary testicular lymphoma (~73%), primary breast lymphoma (~56%), primary intravascular lymphoma (~44%), and primary cutaneous lymphoma (~83%) (40). In ABC DLBCL, MYD88L265P frequently co-occurs with mutations in CD79B and this co-occurrence is even more pronounced in primary extranodal large cell lymphomas highlighting the convergence of BCR and TLR signaling in ABC-DLBCL (27, 40). In a phase 1/2 clinical trial involving 80 subjects with RR DLBCL, use of the BTK inhibitor ibrutinib led to complete or partial responses in 37% (n=14/38) of subjects with ABC DLBCL suggesting that BTK inhibition is insufficient therapy for ABC-DLBCL (41). In ABC DLBCL cell lines with the MYD88 L265P mutation, knockdown of MYD88, IRAK-1, or IRAK-4 (via RNAi) inhibited NF-kB activation and resulted in rapid apoptosis of the cell lines highlighting that sustained MYD88-IRAK pathway signaling is essential for ABC DLBCL cell growth and survival (27). In a mouse xenograft model of ABC DLBCL, ND-2158 (an IRAK-4 inhibitor) decreased tumor growth without excess toxicity and significantly reduced phosphorylation of IRAK-4 (at Thr-345/Ser-346) within the tumors (42). Inhibition of IRAK-4 decreases multiple biological responses downstream of oncogenic MYD88 signaling in ABC DLBCL (42).

### 2.2 Chronic lymphocytic leukemia

In CLL, as opposed to DLBCL, activation of BCR signaling does not involve activating BCR pathway mutations and can thus be viewed as antigen-dependent, resulting from BCR ligation via antigens that are present in the tumor microenvironment (43). Mutations in the MYD88/TLR pathway are rare in CLL but have been identified in patients with CLL (29, 44, 45). Despite the lack of BCR activating pathway mutations in CLL, BTK inhibition is a highly effective therapeutic strategy. Also, despite the low incidence of MYD88/TLR pathway mutations in CLL, TLR pathway inhibition is a viable therapeutic strategy for CLL as preclinical data has shown that inhibition of IRAK-4 with a competitive IRAK-4 inhibitor (ND2158) decreased the viability and proliferation in patient-derived CLL cells, including those that did not harbor MYD88L265P mutations suggesting that IRAK-4 promotes tumorigenesis independent of MYD88L265P mutation (46, 47).

### 2.3 Waldenstrom macroglobulinemia/lymphoplasmacytic lymphoma

Mutations in BCR occur less frequently in WM and are limited to mutations in the CD79A and CD79B genes, in about 15% of WM cases (48, 49). Nonetheless, WM cells exhibit constitutive activation of BCR-related signaling elements (50). 90% of WM/LPL patients harbor MYD88L265P mutations (22). In WM patient samples, BTK has been shown to complex with MYD88 in MYD88L265P-expressing WM cells, with preferential binding to phosphorylated BTK (pBTK). Increased pBTK has been observed in WM cells transduced to overexpress L265P vs wild-type MYD88 and treating these MYD88L265P-mutated WM cells with a BTK inhibitor abrogated MYD88 binding to pBTK (51). These studies highlight the role of BTK in TLR signaling in WM. However, complete response rates with single-agent BTK inhibitors in Waldenström’s Macroglobulinemia are low suggesting that alternative survival signaling pathways in WM cells remain activated in patients treated with BTK inhibitors (33). Phospho-flow analysis of bone marrow WM cells obtained from WM patients following greater than 6 months of continued BTK inhibition with ibrutinib demonstrated highly active IRAK-1 and -4 kinases, but not BTK (39). Additionally, WM cells lines with the MYD88L265P mutation treated with ibrutinib and an IRAK-4/IRAK-1 inhibitor had more robust reductions in NF-kB signaling, and greater amounts of WM cell death compared to each agent alone (39, 51).

### 2.4 Primary CNS lymphoma

A study evaluating PCNSL samples from newly diagnosed human subjects revealed that 68 out of 71 PCNSLs had mutations in genes affecting NF-kB signaling; these mutations affected CD79B (83%) and MYD88 (76%) most predominantly (52). The MYD88L265P mutation in particular is found in 56% of all PCNSLs (40). As BTK plays a role in both BCR and TLR signaling, the therapeutic strategy of BTK inhibition is effective for patients with PCNSL as a phase I trial of ibrutinib monotherapy in PCNSL patients produced an overall response rate of 77% (n=10/13) including 5 complete responses (53). However, this strategy is not curative as at a median follow-up of 479 days, the median PFS was 4.6 months and the median OS was 15 months. Given the high rates of MDY88L265P mutations in PCNSL, it is plausible that TLR signaling inhibition may also be an efficacious therapeutic strategy. In a PCNSL xenograft model, treatment with 100mg/kg of the IRAK-4 kinase inhibitor emavusertib improved median survival by 68%, with 37.5% (n=3/8) of mice experiencing durable survival outcomes. Similar results were observed in syngeneic BALB/c mice harboring A20 primary CNS lymphoma tumors, where 100mg/kg dosing of emavusertib treatment improved median survival by 61% (54).

### 2.5 Marginal zone lymphoma

Chronic antigen stimulation leads to constitutive BCR activity in many marginal zone lymphomas (MZL) (55). A study which used PCR and DNA sequencing to identify somatic mutations in 57 cases of splenic MZL found mutations in MYD88 (6/46 = 13%), A20 (6/46 = 13%), CARD11 (3/34 = 8.8%), but not in CD79A or CD79B (56). As CARD11 acts as a scaffold linking BCR signaling to the NF-kB pathway, MZLs have activating mutation of the BCR pathway and as MYD88 mutations are also seen in MZLs, TLR signaling is also active. Given BTK’s role in both BCR and TLR signaling, BTK inhibition with ibrutinib in relapsed/refractory MZLs resulted in a median PFS of 15.7 months, an ORR of 58%, a median duration of response (DOR) of 27.6 months, and median OS was not reached (57). With regards to TLR signaling inhibition, pre-clinical data has shown that in MZL cell lines, the IRAK-4 inhibitor emavusertib, is synergistic with drugs that inhibit BCR signaling pathways such as ibrutinib and idelalisib (58). Investigators evaluated IRAK-4 inhibition with emavusertib against a panel of MZL cell lines that had developed resistance to PI3 Kinase inhibitors idelalisib and copanlisib or the BTK inhibitor ibrutinib. In resistant cells, emavusertib was strongly synergistic with idelalisib, ibrutinib, and to a lesser extent, with copanlisib. Emavusertib in combination with ibrutinib was synergistic especially in the ibrutinib resistant cell line. Emavusertib (from 1 to 5 µM) restored sensitivity to ibrutinib at IC50 values close to the parental, ibrutinib-resistant cell line. Similarly, emavusertib in combination with idelalisib was also synergistic and increased sensitivity to idelalisib in idelalisib resistant cells (58).

### 2.6 Mantle cell lymphoma

Phosphorylation of several kinases downstream of the BCR such as SYK, LYN and BTK have been noted in primary mantle cell lymphoma (MCL) cells, suggesting constitutively activated BCR signaling in MCL (59). Additionally, amplification of SYK and the PI3K catalytic subunit are found in a subset of MCL which is also evidence of constitutively activated BCR signaling in MCL (60, 61). A phase 2 study of the BTK inhibitor ibrutinib involving patients with relapsed or refractory MCL showed a median PFS of 13.9 months, an ORR of 68%, and a CR rate of 21% (62). However, BTK inhibition is not curative with most patients eventually experiencing relapsed disease while on treatment. MCL cells express many different TLRs with TLR4 being one of the most highly expressed and lipopolysaccharide-induced TLR 4 signaling has been shown in MCL cell lines and MCL patient cells (63). TLR inhibition with the IRAK-4 inhibitor emavusertib has shown efficacy in patients with MCL (64).

The above-mentioned data indicate that IRAK-4 inhibition is an efficacious therapeutic strategy for B-cell lymphomas. Furthermore, IRAK-4 inhibition can synergize with drugs that inhibit BCR signaling pathways such as PI3K and BTK inhibitors to augment anti-lymphoma activity. The development of IRAK-4-selective kinase inhibitors for the treatment of B-cell malignancies with active TLR signaling is warranted.

## 3 TLR signaling in myeloid malignancies

The oncogenic long form of IRAK-4 (IRAK4-L) has been found to be overexpressed in acute myeloid leukemia (AML) and myelodysplastic syndromes (MDS) and portends a worse prognosis (6, 65). An analysis of exon usage in MDS/AML cell lines revealed an enrichment of genes associated with inflammatory pathways regulated by isoform changes in RNA and that isoform expression of IRAK-4 was the most significantly altered (6). This isoform encodes IRAK4-L resulting in “myddosome” activation and subsequent NF-kB activation and leukemic cell survival (6). In particular, MDS/AML cell lines with mutations in the splicing factors U2AF1 and SF3B1 had higher expression of IRAK4-L and inhibition of IRAK-4 abrogated leukemic growth more effectively (65). Hypomethylating agents such as decitabine and azacitidine with or without the BCL-2 inhibitor venetoclax rarely lead to long-term disease control in myeloid neoplasms (41), thus the addition IRAK-4 inhibition may deepen and prolong responses in patients with myeloid malignancies. This preliminary data also supports the evaluation of IRAK-4 inhibition for the treatment of myeloid neoplasms, particularly if they harbor mutations in splicing factors.

## 4 IRAK 4 inhibition: emavusertib

Emavusertib (CA-4948) is an orally bioavailable, selective small molecule inhibitor of IRAK-4 with an IC50 of 57 nM in a fluorescence resonance energy transfer (FRET) kinase assay (66). Emavusertib was discovered via a kinase focused library screening in which the cocrystal structure of an early benzoxazole amide hit in complex with IRAK4 revealed the binding mode and critical active-site interactions of benzoxazole amide hit compounds with IRAK-4 (67). Through a series of modifications to optimize binding affinity for IRAK-4, several benzoxazole amide compounds evolved into aza-benzoxazole molecules and continued modifications to optimize aqueous solubility, lipophilicity, permeability, oral pharmacokinetics, and *in vitro* inhibition of IRAK-4 led to the discovery of emavusertib (67). In a competition binding assay, emavusertib exhibited >350-fold higher binding affinity for IRAK-4 than that observed for IRAKs 1, 2 and 3 (66). Besides IRAK-4, emavusertib was also identified to have high binding activity for the receptor-type tyrosine kinase FLT3 and FLT3 variants with single internal tandem duplications (ITD), single kinase domain (KD) mutations, or double ITD/KD mutations (66).

In ABC DLBCL cell lines, IL-6 and IL-10 cytokine production depends on MYD88-mediated NF-kB activation via interaction with IRAK-4 (27). Emavusertib was tested for blockade of IL-6 and IL-10 secretion in 2 ABC and 1 germinal center B-cell (GCB) DLBCL cell lines. Compared to the control of dimethyl sulfoxide (DMSO), emavusertib repressed IL-6 secretion by 36% in 1 of the ABC cell lines. Emavusertib repressed IL-10 secretion in all 3 cell lines tested (by 40% and 41%, respectively, in the 2 ABC cell lines, and by 35% in the GCB DLBCL cell line). Emavusertib’s ability to inhibit the TLR signaling pathway was evaluated in a monocytic cell line using western blot analysis. Inhibition of the TLR pathway was demonstrated via reduced phosphorylation of the downstream proteins I-KappaB kinase (IKKB), NF-kB p65 and extracellular-signal regulated kinase (ERK) in the presence of emavusertib (66). In separate experiments also performed using this cell line, emavusertib reduced levels of an NF-kB reporter gene in cell culture, the expression of which was driven up by TLR-agonist stimulation of NF-kB family members p65 and c-Rel. Furthermore, emavusertib blocked IL-1R stimulated cytokine production in these cells (66). In MZL cell lines, emavusertib treatment (10 μM) for 72 hours decreased the percentage of proliferating cells and induced a moderate increase in the sub-G0 fraction as evaluated by flow cytometry (68). Emavusertib (10 μM, 72 h) induced a significant increase in the apoptotic cell population, particularly when combined with ibrutinib compared to ibrutinib and emavusertib alone. Decreased viability upon treatment with emavusertib was paired with a significant increase in apoptotic cells in both ibrutinib sensitive and ibrutinib resistant MZL cell lines (68).Thus, the mechanism of action of emavusertib is its binding and inhibition of IRAK-4 resulting in blockade of the MYD88 signaling pathway. By inhibiting the pathway, proinflammatory and other growth-related pathways are repressed leading to apoptosis of cells (66, 69).

The *in vivo* efficacy of emavusertib in B-cell NHL was first evaluated in an OCI-Ly3 xenograft model (xenograft model of ABC DLBCL with MYD88*-*L265P mutation) through once daily oral administration. A dose of 200 mg/kg qd showed partial tumor regression and a 100 mg/kg qd dose showed >90% tumor growth inhibition (67, 70). Emavusertib was well-tolerated, and there were no overt toxicities at these efficacious doses. Emavusertib induced 70%, 70% and 54% tumor growth inhibition, respectively, in the ABC DLBCL models LY2345, LY2264 and LY2298 (66). 50 mg/kg and 150 mg/kg of emavusertib qd showed 25% and 70% tumor growth inhibition, respectively, in the LY2264 model (66). A preliminary pharmacodynamics study measuring ex-vivo toll-like receptor induced cytokine release following emavusertib administration in mice suggested there was a potent pharmacodynamic effect of emavusertib after administration of a single dose, which lasted for several hours but returned to baseline before 24 hours post-dose (66). A more frequent dosing schedule of emavusertib was hypothesized to result in a more sustained pharmacodynamic effect, which may lead to enhanced antitumor activity. *In vivo* efficacy studies were conducted in mouse xenograft DLBCL tumor models comparing QD versus BID dose schedules. Mice bearing OCI-LY10 tumors (ABC DLBCL subtype, MYD88-L265P) were orally administered emavusertib either once daily at 25, 50, or 150 mg/kg, or twice daily at 12.5, 25, or 50 mg/kg for 14 consecutive days. The results of this 14-day efficacy study show that at the lowest daily cumulative dose set tested, emavusertib administered as a twice-daily divided dose was equivalent to the corresponding once-daily dose with regards to antitumor activity, i.e., 12.5 mg/kg BID versus 25 mg/kg QD. Furthermore, although not statistically significant (p=0.06, t-test), improved antitumor activity was observed for the BID dose schedule at the next highest daily cumulative dose set tested, i.e., 25 mg/kg BID versus 50 mg/kg QD.

The *in vivo* efficacy of emavusertib on leukemic tumor burden was evaluated in the human THP-1 monocytic (AML, FLT3-wt) cell line model (66). THP-1 cells were systemically injected into mice via tail-vein injection and animal survival and degree of AML cell engraftment were monitored in 100 mg/kg emavusertib-treated mice compared with vehicle-treated control mice. In the survival study, emavusertib was dosed for the first 47 days after which dosing was stopped and the animals were monitored for survival (66). The vehicle-treated animals rapidly and uniformly succumbed on day 60 while the survival of the emavusertib treated mice was significantly extended for an additional 10 days (66). Examination of the mouse bone marrow after 41 or 44 days of emavusertib treatment showed a nearly complete absence of THP-1 cell engraftment while the bone marrow of vehicle-treated animals consisted of 15% - 45% THP-1 cells (66). The effect of emavusertib on tumor growth was evaluated *in vivo* in the subcutaneous MV4-11 and MOLM-14 AML tumor models which harbor FLT3-ITD mutations and are sensitive to FLT3 inhibitors in cell-based assays and subcutaneous tumor xenograft model studies (71). The results from two independent MV4-11 xenograft studies demonstrated induced tumor regression during the 21-day treatment period with 12.5, 25, 50 and 100 mg/kg of emavusertib administered via oral gavage. A complete tumor regression response was maintained for more than 60 days post treatment in the 100 mg/kg treated group (66).

The results of preclinical studies with emavusertib demonstrate potent inhibition of TLR signaling and subsequent inhibition of proinflammatory and cellular proliferation pathways in both B-cell NHL and myeloid malignancies. Additionally, emavusertib showed antitumor efficacy with an acceptable toxicity profile in mouse models of B-cell NHL and AML. The evaluation of emavusertib in early phase clinical trials for the treatment of relapsed and refractory B-cell and myeloid malignancies is merited given the promising pre-clinical efficacy and safety of emavusertib.

## 5 Emavusertib for the treatment of non-Hodgkin’s lymphoma

A multicenter, phase I/II, open-label clinical trial (with an estimated/planned enrollment of 221 patients) is evaluating the administration of emavusertib (CA-4948) in patients with relapsed/refractory B-cell NHL (NCT03328078). Part A of this clinical trial will evaluate escalating doses of emavusertib monotherapy (Part A1) or emavusertib in combination with ibrutinib for B-cell NHL (Part A2). Once the recommended phase 2 dose (RP2D) of emavusertib and ibrutinib has been determined, Part B will consist of a safety expansion phase to assess the efficacy (complete response rate or overall response rate) and safety of the RP2D of emavusertib and ibrutinib in four B-cell NHL disease-specific cohorts; MZL (cohort 1), ABC DLBCL or extranodal subtypes Leg-, testicular-, or not otherwise specified (NOS)-type (cohort 2), PCNSL (cohort 3), patients receiving ibrutinib monotherapy who have developed secondary resistance, and indications for which ibrutinib is National Comprehensive Cancer Network-listed (cohort 4).

The first report of the phase 1a, dose-escalation portion of this clinical trial evaluated the safety and pharmacokinetics of emavusertib at doses varying between 50mg PO qd and 400mg PO BID in 22 patients with various B-cell NHLs; 13 DLBCLs, 5 follicular lymphomas (FL), 1 high grade b-cell lymphoma, 1 MCL, 1 WM, and 1 LPL. The most common treatment emergent adverse events (TEAE’s) unrelated to emavusertib were: fatigue (36%), nausea (27%), neutrophil count decreased (23%), dizziness (18%), hypercalcemia (18%), hypophosphatemia (18%), and vomiting (18%). Eleven patients (50%) developed grade 3/4 TEAEs considered related to emavusertib, and 4 patients experienced serious adverse events, one of which was considered related to emavusertib: grade 3 rhabdomyolysis (72). An update of the phase 1a presented at the American Society of Hematology Annual meeting in December 2020 included data from 30 patients (as of July 2020) with relapsed or refractory NHL, including DLBCL (n=14), FL (n=6), transformed high grade B-cell lymphoma (n=1), WM (n=3), LPL (n=2), MZL (n=2) and MCL (n=2). The median age was 68.5 (range 50 - 87;83% male) and the median number of prior therapies was 4 (range 1 – 8). Prior therapies included chimeric antigen receptor (CAR) T-cell treatments or autologous hematopoietic cell transplantation in 5 patients. The RP2D was 300mg BID due to grade 3 rhabdomyolysis which represented a dose limiting toxicity in two of eight patients treated with 400mg BID of emavusertib. The duration of treatment ranged between 15 days and 19+ months (with continued disease control). Eight of 28 evaluable patients experienced overall tumor burden decreases of ≥20% from baseline. A heavily pretreated WM patient with 6 prior lines of therapy, sustained a partial response and underwent intra-patient dose escalation and had a dose/response relationship without dose limiting toxicity. The median duration of treatment at doses of 200 or 300 mg bid was >6 months (range 1->19)) (64). Phosphorylation of NF-kB-p50 has been identified as a possible biomarker for response to emavusertib. In tumor biopsy samples obtained from 14 patients with B-cell NHLs prior to treatment with emavusertib, analysis of NF-kB phospho-p50 S337 expression revealed nuclear and/or cytoplasmic expression of NF-κB-p50 in 85% (n=6/7) of stable disease cases treated with 50mg QD (n=3), 50mg BID (n=1), 200mg BID (n=1) and 400mg BID (n=2). NF-kB-p50 expression was not detected in 85% (n=6/7) of cases with progressive disease including patients treated with 50mg QD (n=1), 100mg QD (n=1), 100mg BID (n=3), 200 mg BID (n=1) and 400mg BID (n=1). p-IRAK-1 expression in tumor biopsies was not correlated with clinical responses (73).

In Part A2 of this clinical trial, 13 patients were treated with emavusertib (200mg BID or 300mg BID) + ibrutinib (420mg or 560mg QD). The median number of prior therapies was 3 (range 1-8) and the median age was 66 years (range 56-92). Two patients had CLL, 2 patients had PCNSL, 2 patients had DLBCL, 2 patients had MCL, 3 patients had MZL and 2 patients had WM. Six of these 13 patients had been previously treated with a BTK inhibitor. Nine patients were evaluable for response; 4/13 patients were not evaluable for response because 1 patient progressed without measurable tumor burden and 3 patients did not have a response assessment prior to discontinuation of therapy (1 adverse event, 1 death, 1 other). The ORR was 44.4% (n=4/9) including two complete responses (a PCNSL and an MCL) and two partial responses (an MCL and a CLL). The clinical benefit rate was 100% with 5 additional patients achieving stable disease. Of the evaluable patients previously treated with a BTK inhibitor (n=4), 1 of the 4 (25%) patients achieved a response (a PCNSL who achieved a CR) and 3 achieved SD. There were no observed dose limiting toxicities at 200mg but 2 dose limiting toxicities were observed at the 300 mg (stomatitis and syncope) dose level to date. Presently, all responding patients are receiving 200mg BID of emavusertib with full dose of ibrutinib. This data highlights the acceptable long term safety profile and promising efficacy of the combination of emavusertib and ibrutinib (74). Additionally, this data supports the combination of BTK inhibition and IRAK-4 inhibition for augmented inhibition of myddosomal signaling that leads to anti-lymphoma activity even in the setting of prior BTK exposure and/or refractoriness. Clinical trial results of emavusertib in B-cell NHL are summarized in **Table 1**.

**Table 1:** Clinical trials evaluating emavusertib in non-hodgkin lymphomas.

Clinical Trials.gov Identifier	Diseases Evaluated	Median Age, range	Median # of Prior Treatments, range	Any Grade Treatment Related Adverse Events	≥Grade 3 Treatment Related Adverse Events	Response	RP2D
NCT03328078	**Phase 1a** **(Emavusertib monotherapy)** DLBCL (n=14) MCL (n=2) MZL (n=2) WM (n=3) Transformed DLBCL (n=1) LPL (n=2) FL (n=6)	68.5 (50-87)	4 (1-8)	Fatigue (36%), Nausea (27%), Neutrophil count decreased (23%), Dizziness (18%), Hypercalcemia (18%), Hypophosphatemia (18%), Vomiting (18%).	Rhabdomyolysis (25%; n=8) of patients treated with 400mg BID	Eight of 28 evaluable patients experienced overall tumor burden decreases of ≥20% from baseline	300mg BID of Emavusertib
	**Part 2a (Emavusertib + Ibrutinib 420mg or 560mg)** DLBCL (n=2) CLL(n=2) MCL (n=2) MZL (n=3) WM (n=2) PCNSL(n=2)	66 (56-92)	3 (1-8)	NR	NR	ORR=44.4% (n=4/9) CR=2 (PCNSL and MCL) PR=2 (MCL and CLL) 5=SD	200mg BID of Emavusertib combined with Ibrutinib 420 or 560mg

DLBCL, diffuse large B cell lymphoma; MCL, mantle cell lymphoma; MZL, marginal zone lymphoma; WM, Waldenstrom macroglobulinemia; LPL, lymphoplasmacytic lymphoma; FL, follicular lymphoma; CLL, chronic lymphocytic leukemia; PCNSL, primary CNS lymphoma; NR, not reported, ORR, overall response rate; CR; complete response; PR; partial response; SD, stable disease; RP2D, recommended phase II dose.

#, Number.

## 6 Emavusertib pharmacology

**Figure 2:**
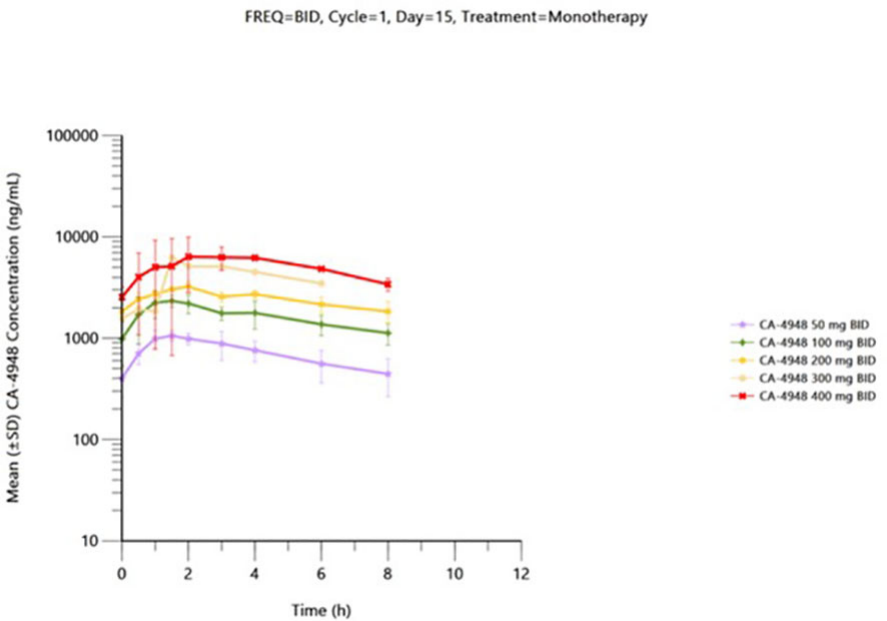
Mean (± SD) plasma CA-4948 concentration-time plot by dose on cycle 1 day 15 following BID CA-4948 monotherapy administration - (semi-log scale) – pharmacokinetic population.

Pharmacokinetic data is available from 41 patients received the first dose of emavusertib and were evaluable on Day 1. Thirty-one patients received emavusertib as a BID regimen and of those, 9 patients received emavusertib in combination with ibrutinib. Ten patients received emavusertib as a QD regimen with only 1 of those patients receiving emavusertib in combination with ibrutinib. The results for single and multiple dose are described below for the BID regimen of emavusertib. Following a single dose of emavusertib in the BID regimen as a single agent, geometric mean Cmax was 893 ng/mL, 1770 ng/mL, 2100 ng/mL, 4000 ng/mL and 7080 ng/mL with moderate to high geometric CV% ranging from 32.7% to 101% for 50 mg, 100 mg, 200 mg, 300 mg and 400 mg BID groups respectively. Median t_max_ ranged from 1.45 to 3.00 hours post dose for the 50 mg to 400 mg BID single agent groups. Geometric mean AUC_0-8_ was 3820 h*ng/mL, 9310 h*ng/mL, 14000 h*ng/mL and 32300 h*ng/mL with low to moderate geometric CV% ranging from 26.8% to 95.5% for 50 mg, 100 mg, 200 mg and 400 mg BID groups respectively. Exposures of emavusertib increased with increasing dose in an approximate dose proportional manner, with an 8-fold increase in dose (50 mg to 400 mg), a 7.9- and 8.5-fold increase was observed in C_max_ and AUC_0-8_ exposures, respectively. Following multiple BID administrations of emavusertib as a single agent, median C_max_ was 1140 ng/mL (n=2), 2640 ng/mL (n=3), 3300 ng/mL (n=2), 6240 ng/mL (n=1) and 6000 ng/mL (n=1) for 50 mg, 100 mg, 200 mg, 300 mg and 400 mg BID respectively. Individual t_max_ values ranged from 0.50 to 4.00 hours post dose. Median AUC_0-8_ were 5730 h*ng/mL (n=2), 13100 h*ng/mL (n=3), 19700 h*ng/mL (n=2) and 35600 h*ng/mL for 50 mg, 100 mg, 200 mg and 400 mg BID respectively. AUC_0-8_ could not be determined for the 300 mg BID patient. Mean accumulation values (n > 2) of emavusertib C_max_ and AUC_0-8_ ranged from 1.32 to 1.63 and 1.59 to 1.75. Overall, Exposures (C_max_ and AUC_0-8_) increased with increasing dose in an approximate dose proportional manner following single administration of emavusertib on Day 1 and a slightly less than dose proportional manner following multiple BID dosing on Day 15 (**Figure 2**). Low to moderate accumulation (~ 1.32 to 1.75) of emavusertib is seen following repeat BID administration of emavusertib and does not appear dose dependent (unpublished data).

## 7 Emavusertib for the treatment of primary central nervous system lymphoma

Emavusertib is also showing efficacy in patients with PCNSL. In initial proof-of-concept studies leveraging preclinical murine models of PCNSL, ultraperformance liquid chromatography-tandem mass spectrometry (LC-MS/MS) analysis of cerebrospinal fluid (CSF) and brain tissue was performed to evaluate whether emavusertib could demonstrate blood-brain barrier penetrance. Emavusertib concentration was measured at set time points following administration of a single dose of emavusertib. Time to maximum concentration (Tmax) was similar in both CSF (0.25h) and brain parenchyma (0.5h) compared to plasma (0.38 ± 0.14h). There was a slight delay in the brain parenchymal accumulation of emavusertib in mice harboring A20 PCNSL tumors (0.83 ± 0.29h). The plasma half-life (T1/2) was 2.73h which was approximately 2x the T_1/2_ found in naïve mice CSF (1.33h), brain parenchyma (1.39h), and tumor-bearing brain parenchyma (1.19h). These results suggest that emavusertib is cleared more rapidly from the CNS than the plasma. While brain concentrations of emavusertib were lower as compared to plasma- 1.53%, 4.26%, and 4.95% for naïve CSF, naïve brain parenchyma, and tumor-bearing brain parenchyma, respectively, emavusertib concentrations in all of the CNS compartments exceed the proliferative IC50 values established for each OCI-LY3 and A20 lymphoma mouse models indicating emavusertib’s ability to achieve therapeutic dose levels in the CNS. In mice harboring the ABC DLBCL tumor OCI-LY3, treatment with 100mg/kg of emavusertib led to a 68% improvement in median survival, with 3 of 8 mice experiencing durable survival outcomes. Similar results were observed in mice harboring A20 PCNSL tumors, where treatment with 100mg/kg of emavusertib improved median survival by 61% (54). Emavusertib reduced MAP kinase and NF-kB expression in a preclinical model of PCNSL. Together these data support that emavusertib is capable of pharmacologically targeting IRAK-4 in the CNS.

Two patients on clinical trial (NCT03328078) received ibrutinib 560mg QD in combination with emavusertib 300mg BID. One patient was a 66-year-old female with PCNSL who had received 2 prior lines of therapy, most recently Ibrutinib, and achieved complete remission with the combination of ibrutinib and emavusertib. The other patient was a 65-year-old male with DLBCL with secondary CNS involvement who had received 4 prior lines of therapy and was ibrutinib naïve who achieved stable disease and resolution of neurologic symptoms for 5 months with ibrutinib and emavusertib. No dose-limiting toxicities or treatment-related adverse events were reported. One patient developed grade 3 thrombocytopenia, pain and muscular weakness which eventually resolved and did not require dose adjustment. The other patient developed grade 3 hyperbilirubinemia, AST and ALT increases which resolved with dose holds and did not reoccur with re-challenge (75). This data supports the promising efficacy of emavusertib in combination with BTK inhibition for the treatment of CNS lymphomas.

## 8 Emavusertib for the treatment of myeloid malignancies

**Figure 3:**
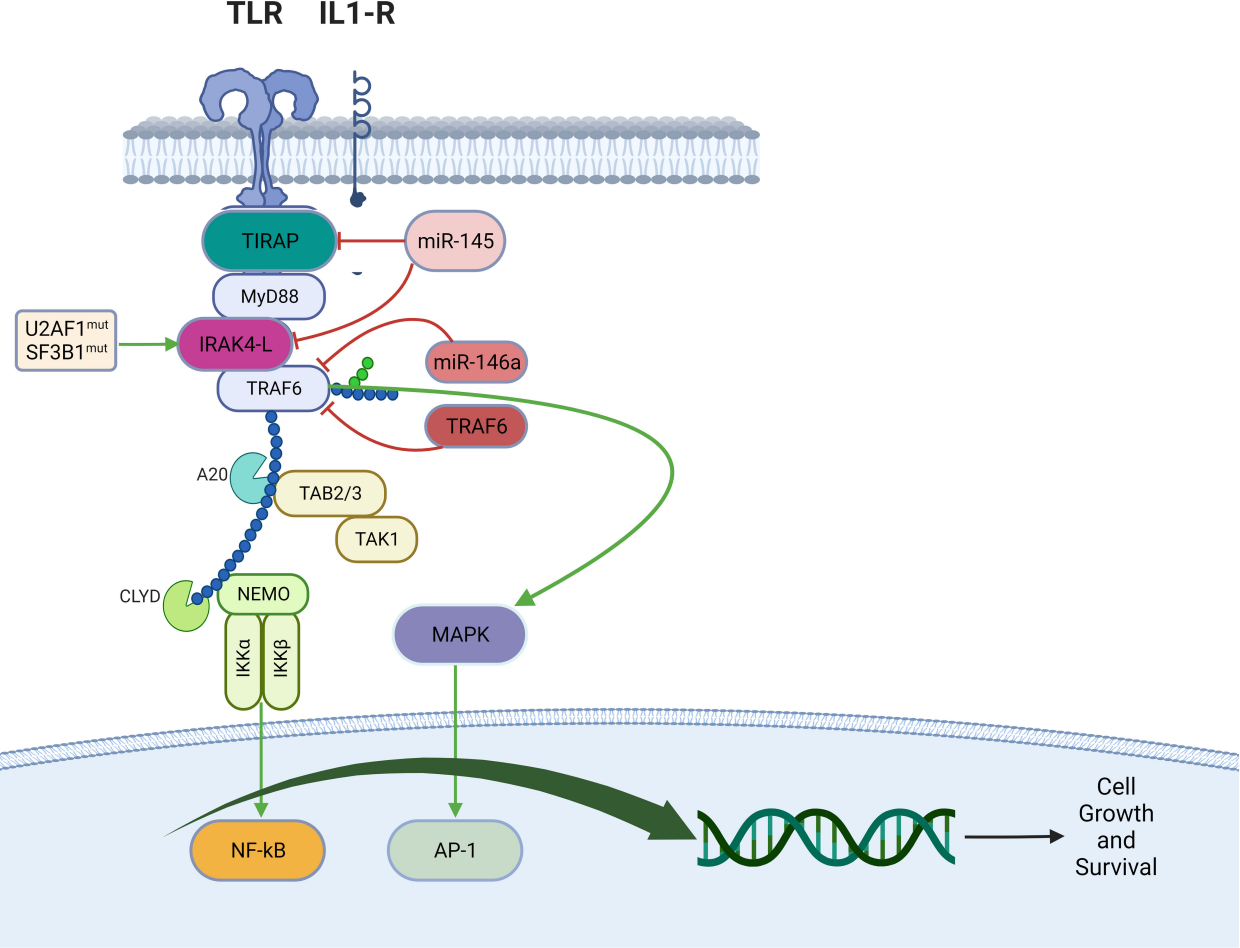
Myddosomal signaling in myeloid malignancies.

In AML and MDS, there is aberrant interleukin 1 receptor-associated kinase-dependent signaling (**Figure 3**). Receptors such as TLRs and IL-1R recruit adaptor proteins TIRAP and MYD88 together with the IRAK-1/4 kinases and TRAF6, to form the “myddosome complex”. miR-145, miR-146a, and TIFAB, negative regulators of the myddosome pathway, are often deleted in MDS and AML (76, 77). Mutations in the spliceosome proteins U2AF1 and SF3B1 convert hypomorphic (IRAK4-S) to hypermorphic (IRAK4-L) IRAK-4 isoforms and the hypermorphic IRAK-4-L recruits MYD88 and IRAK-1 to activate downstream NF-kB and MAPK signaling (78). Emavusertib showed antileukemic activity both in FLT3-wild type and FLT3-mutated mouse models of AML. MOLM-14 double FLT3-ITD/KD, MOLM-14 FLT3-ITD, and MV4-11 AML tumor models were treated with emavusertib and emavusertib demonstrated >500-fold selectivity for IRAK-4 over IRAK-1, a 23 nM (Kd) binding affinity for IRAK-4, and high binding affinity (8–31 nM [Kd]) for FLT3- wt, -ITD, -ITD/D835 V, and -ITD/F691L. Consistent with emavusertib’s ability to inhibit FLT3 activity, emavusertib displayed cytotoxic activity against FLT3 mutated AML cell lines *in vitro* at 58-200 nM (IC50). In *in vivo* studies with FLT3 wt AML tumor models, emavusertib blocked bone marrow engraftment of the THP 1 AML cell line, while the FLT3 inhibitors midostaurin and quizartinib did not. In AML FLT3-ITD tumors, emavusertib induced cytotoxic activity equivalent to quizartinib and midostaurin. Emavusertib also induced cytotoxic activity in FLT3-ITD/D835Y tumors similar to midostaurin and showed greater cytotoxic activity in FLT3-ITD/F691L tumors as compared to quizartinib (79). These results demonstrate that targeting TLR signaling with the IRAK-4 inhibitor emavusertib may be an effective therapeutic strategy in AML with and without FLT3 mutations.

A phase 1/2a dose escalation and dose expansion study (NCT04278768) of emavusertib monotherapy and in combination with azacitidine or venetoclax in adult patients with AML or high-risk MDS is currently enrolling patients at the time of this review article. In a 3 + 3 design, patients with relapsed or refractory AML or high-risk MDS, will receive emavusertib at doses of 200, 300, 400, and 500 mg BID continuously in 28-day cycles until intolerance or progressive disease. In the phase I portion of the trial, the primary objective is safety and the RP2D of emavusertib. Patients must have received between 1-2 prior therapies and have relapsed/refractory AML with FLT3 mutations or with spliceosome mutations of SF3B1 or U2AF1 or relapsed/refractory high risk MDS with mutations of SF3B1 or U2AF1. In the first report from this trial, as of February 2021, 15 patients (5 female) with a median number of prior therapies of 2 (range 1-4) and a median age of 72 years (range: 32-84) had been enrolled. Seven patients had AML and 8 had high risk MDS by IPSS-R. At baseline, most patients were dependent on transfusion support. Dose levels of 200, 300, and 400 mg bid have completed accrual for DLT evaluation. With ongoing enrollment, treatment duration ranged from <1 to 7 months with no DLT reported at the first 3 dose levels up to 400mg BID. In patients with elevated bone marrow blasts, reductions in bone marrow blasts compared to baseline were seen at all dose levels assessed in 89% (n=8/9) of evaluable patients. In responding patients, responses included 1 CR, 2 bone marrow CRs and 1 CRi with negative minimal residual disease. In the three patients with spliceosome mutations, all of them achieved a marrow CR or better. Responding patients also saw signs of hematologic recovery (80).

As of December 2021, 43 patients had enrolled who had received a median of 2 (range 1-5) prior lines of therapy. Emavusertib dose levels of 200 to 500 mg BID were evaluated. No DLTs were observed at either 200 mg or 300 mg BID and no ≥grade 4 TRAEs have been reported. Grade 3 rhabdomyolysis occurred with greater frequency at higher dose levels, occurring in 4% (n=1/26) of patients at 300 mg BID, 12% (n=2/17) of patients at 400 mg BID, and 33% (n=1/3) of patients at 500 mg BID. The rhabdomyolysis was reversible and manageable in all patients. The RP2D was determined to be 300 mg BID. Fourteen of the 43 patients had mutations in SF3B1, U2AF1 or FLT3 and in the 5 evaluable AML patients with mutations in SF3B1 or U2AF1, 40% (n=2/5) achieved CR/CRh (1 CR, 1 CRh). In the 7 high risk-MDS patients with spliceosome mutations, 57% achieved marrow CR. Of the 3 FLT3-mutated AML patients, 2 became FLT3-negative and one achieved CR. Amongst the 29 patients without spliceosome or FLT3 mutations, 1 achieved CR and 2 achieved PR. In patients responding to emavusertib, RNA-sequencing on selected samples showed decreases in the relative expression of IRAK4-long isoforms. Phase 1b and Phase 2a are ongoing (81, 82). In human AML cell lines and clinical AML samples from NCT04278768, investigators performed immunohistochemical staining and found nuclear expression of IRAK-4 in leukemic blasts in 9 out of 19 AML cases. In support of these findings, investigators also detected nuclear IRAK-4 protein expression in the AML cell lines THP-1, HL-60 and K562. Using AML bone marrow samples, investigators found that nuclear expression of IRAK-4 in leukemic blasts correlated with NF-kB activation based on the accumulation of NF-kB p-p50 and p-p65 in the nucleus in 9 out of 19 cases. These findings demonstrate that the co-expression of IRAK-4, NF-kB p-p50 and p-p65 in the nucleus of blasts identifies a novel mode of interaction between IRAK-4 and NF-kB in human AML (83). Clinical trial results of emavusertib in myeloid malignancies are summarized in **Table 2**.

**Table 2:** Clinical trials evaluating emavusertib in myeloid malignancies.

Clinical Trials.gov Identifier	Diseases Evaluated	Median Age, range	Median # of Prior Treatments, range	Any Grade Treatment Related Adverse Events	≥Grade 3 Treatment Related Adverse Events	Response	RP2D
NCT04278768	Phase I **(Emavusertib Monotherapy)** AML MDS N=49	NR	2 (1-5)	NR	No Grade 4 or 5 treatment-related adverse events were reported Reversible, manageable Grade 3 rhabdomyolysis occurred in 1/26 (4%) patients at 300 mg BID, 2/17 (12%) at 400 mg BID, and 1/3 (33%) at 500 mg BID	In the 5 evaluable AML patients with spliceosome mutations, 40% reached CR/CRh (1 CR, 1 CRh) In the 7 spliceosome-mutated high risk-MDS patients, 57% reached marrow CR Among the 29 patients without SF3B1/U2AF1/FLT3 mutations, 1 reached CR and 2 PR	300mg BID

AML, acute myeloid leukemia; MDS, myelodysplastic syndrome; NR, not reported; ORR, overall response rate; CR, complete response; CRh, complete response with partial hematologic recovery; PR, partial response; RP2D, recommended phase II dose.

#, Number.

## 9 Conclusion and future directions

The TLR signaling pathway plays a crucial role in cell survival in B-cell and myeloid malignancies. In B-cell malignancies with highly active BCR signaling and/or with MYD88L265P mutation, BTK inhibition leads to objective responses and disease control but rarely are complete responses seen and relapse is inevitable. Persistent TLR signaling downstream of BTK and MYD88 in B-cell NHL patients treated with BTK inhibitors highlight the need to target other proteins in the TLR pathway. Persistent IRAK-4 signaling in the setting of treatment with BTK inhibitors has been shown in B-cell lymphomas making IRAK-4 inhibition an attractive therapeutic target. In myeloid malignancies such as MDS and AML, spliceosome mutations of SF3B1 or U2AF1 lead to formation of hypermorphic IRAK-4 isoforms (IRAK-4-L) which activate myddosomal signaling promoting cell survival. Preclinical studies have shown the anti-B cell NHL and anti-AML/MDS therapeutic efficacy of IRAK-4 inhibition. The orally bioavailable IRAK-4 inhibitor, emavusertib, is emerging as an efficacious treatment in the therapeutic armamentarium against B-cell NHL and MDS/AML. In the Phase I/II TakeAim Lymphoma trial (NCT03328078), emavusertib is showing efficacy in several B-cell NHLs as a single agent and also in combination with the BTK inhibitor ibrutinib, even in patients with prior BTK inhibitor exposure. Emavusertib also crosses the blood brain barrier and is efficacious in patients with PCNSL. The TakeAim Lymphoma trial will shift its focus and will only enroll patients with PCNSL. Additionally, there is a planned clinical trial that will combine the BTK inhibitor zanubrutinib with emavusertib for patients with relapsed/refractory WM. Emavusertib appears to be well-tolerated with rhabdomyolysis being the most significant dose-limiting toxicity. In the phase I/II TakeAim Leukemia trial (NCT04278768), emavusertib has shown efficacy and tolerability in relapsed/refractory AML/MDS with spliceosome mutations.

**Figure 4:**
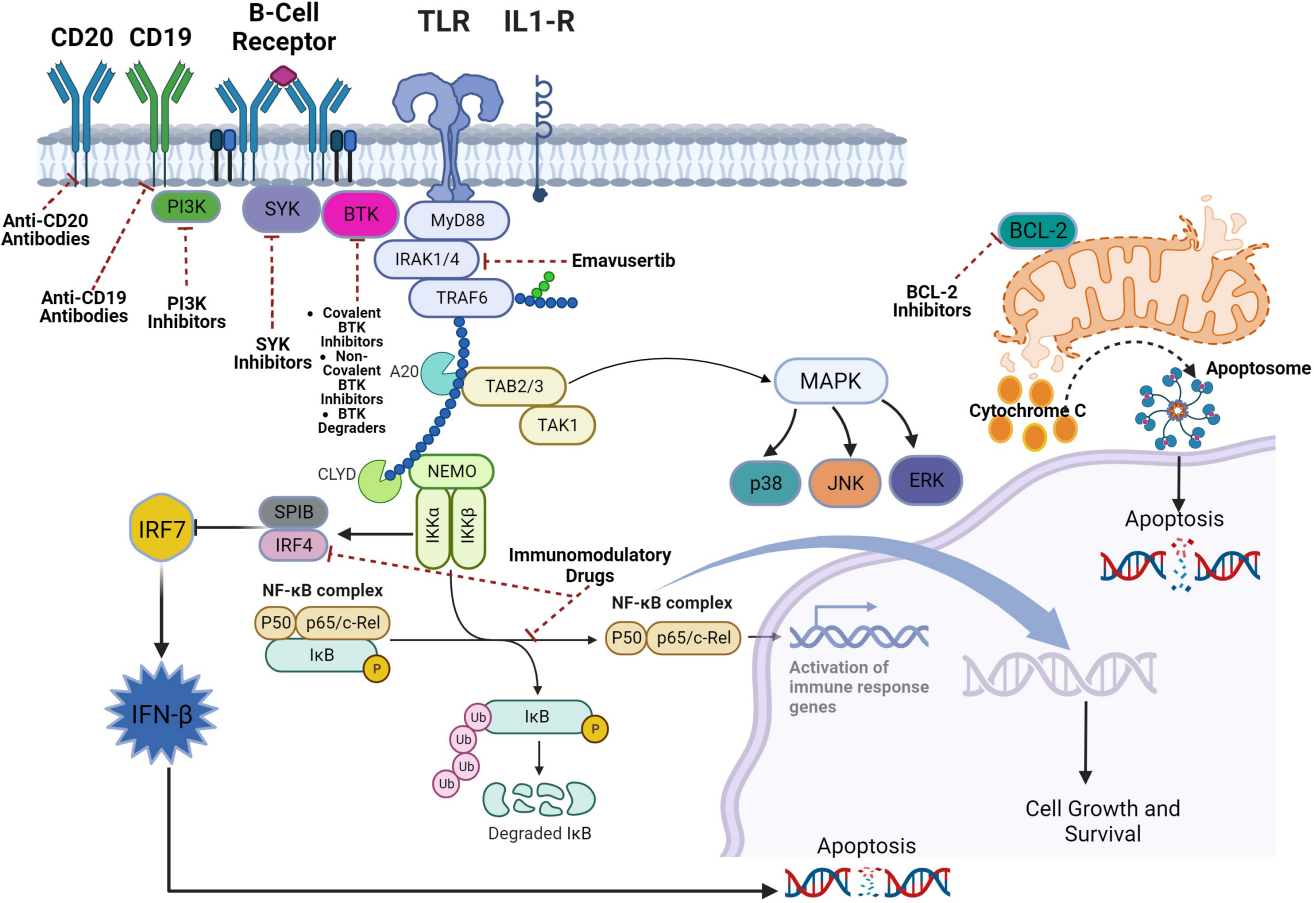
Combining IRAK-4 inhibition with other therapies in B-Cell malignancies.

In B-cell NHL, emavusertib has shown synergy with covalent BTK inhibitors such as ibrutinib and with PI3K pathway inhibitors such as idelalisib and copanlisib (68, 74). However, most patients ultimately develop resistance to covalent BTK inhibitors and PI3K inhibitors (38, 84). While emavusertib has shown efficacy when combined with ibrutinib and PI3K inhibitors even in patients with secondary resistance to these agents (68), there is rationale to combine emavusertib with next generation inhibitors of the BTK pathway such as non-covalent BTK inhibitors and BTK degraders given the impressive efficacy of these agents in several heavily pre-treated B-cell NHL patients that developed disease progression after prior treatment with a covalent BTK inhibitor and even prior PI3K inhibitors (85, 86). Inhibition of additional proteins in the BCR pathway such as SYK have shown pre-clinical efficacy when combined with emavusertib in B-cell NHL (42). Additionally, combining emavusertib with a BCL-2 inhibitor has also shown synergistic levels of apoptosis in an ABC-DLBCL preclinical model (42). Anti-CD20 monoclonal antibodies are part of many therapeutic regimens for the treatment of B-cell NHL, however, there is data suggesting that BCR pathway inhibitors such as BTK, SYK and PI3K kinase inhibitors may impair the efficacy of anti-CD20 monoclonal antibodies via down-regulation of cell surface levels of CD20 and impairment of antibody-dependent cell-mediated cytotoxicity and complement-dependent cytotoxicity (87). As emavusertib mainly targets TLR signaling, it would be merited to evaluate the combination of an anti-CD20 monoclonal antibody with emavusertib in B-cell NHL to see if there is synergism between the two therapies and also the combination of emavusertib with anti-CD19 monoclonal antibodies which have shown single agent efficacy in B-cell NHL (88). Immunomodulatory drugs such as lenalidomide have also shown efficacy in B-cell NHL (89). Signaling molecules IRF4 and SPIB lie at the nexus of the BCR and TLR signaling pathways promoting ABC DLBCL survival by repressing IRF7, blocking detrimental IFNβ signaling, and transactivating CARD11 which in turn promotes NF-κB signaling and survival of ABC DLBCL (90). Lenalidomide down regulates IRF4 and SPIB, thereby increasing toxic IFNβ secretion and decreasing NF-κB activity which promotes apoptosis of ABC-DLBCL cells (90). Combining emavusertib with lenalidomide for the treatment of B-cell NHL makes mechanistic sense given the convergence of the BCR and TLR pathways in many B-cell NHLs. Potential agents that can be combined with emavusertib for RR B-cell NHL are shown in **Figure 4**.

**Figure 5:**
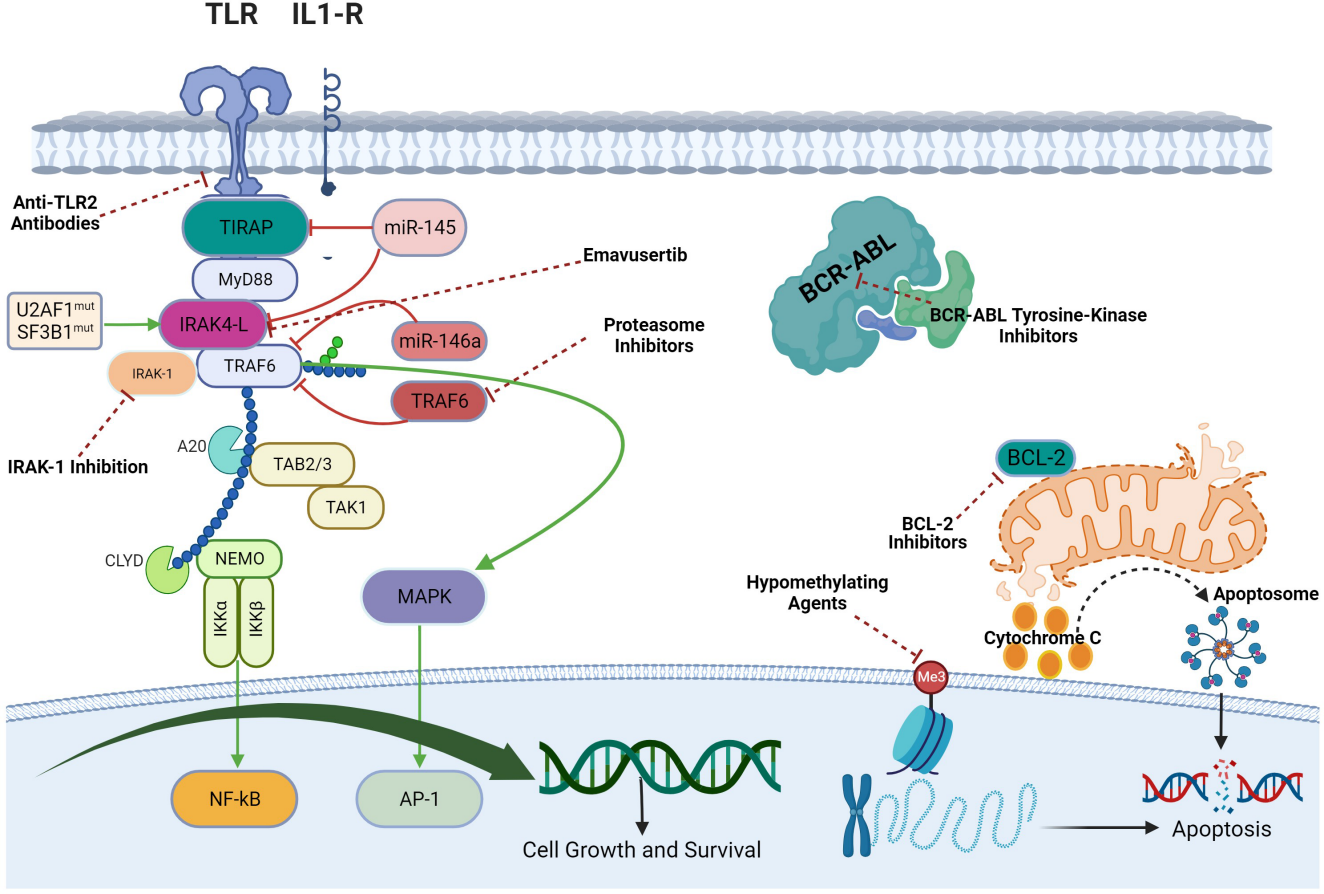
Combining IRAK-4 inhibition with other therapies in myeloid malignancies.

In myeloid malignancies, rationale exists for combining emavusertib with other agents that target the TLR/innate inflammatory pathway as well as the intrinsic apoptotic pathway (2) (**Figure 5**). Augmented TLR signaling inhibition can theoretically be achieved by combining emavusertib with tomaralimab (OPN-305), a fully humanized IgG4 k monoclonal antibody against TLR-2. In a phase 1 trial performed in patients with heavily pre-treated, transfusion-dependent, lower-risk MDS, 50% of patients experienced hematologic improvement and 2 achieved red blood cell transfusion independence with tomaralimab (91). Augmented TLR signaling inhibition can also be achieved by combining emavusertib with a proteasome inhibitor such as bortezomib which via lysosomal degradation, can degrade the TLR-signaling intermediate TRAF6, leading to apoptosis of AML/MDS cells (92). In a phase II trial, bortezomib treatment resulted in hematologic improvement in 20% (3/15) of lower-risk MDS patients, with accompanying reduction in NF-kB signaling (93). While upregulation of BCL2 was noted in MDS/AML clones that escaped IRAK1 inhibitor treatment, synergistic reduction of cell expansion and viability was obtained upon the addition of BCL2 inhibitor to an IRAK1 inhibitor suggesting that there is synergy between TLR signaling blockade and inhibition of anti-apoptotic pathways in myeloid malignancies (94). *In vitro* data has shown that emavusertib potentiates the antitumor effects of azacitidine in 3 of 4 FLT3-wild type AML cell lines whereas there was no additive or synergistic effect of combining emavusertib with decitabine (95). There is also data supporting the dual inhibition of IRAK-1 and IRAK-4 to suppress leukemic stem/progenitor cell function and induce differentiation in cell lines and patient-derived cells (96). In chronic myelogenous leukemia (CML), blocking NF-κB signals with IRAK-4 inhibition and BCR-ABL inhibition has been found to eliminate mouse and human CML leukemic stem/progenitor cells (97).

Mounting pre-clinical and early phase clinical trial data highlight the preliminary safety and efficacy of emavusertib both as a single agent and in combination with other agents in patients in B-cell NHL and myeloid malignancies. The central role of IRAK-4 in aberrant TLR/innate immune signaling in B-cell NHL and myeloid malignancies makes emavusertib a promising therapeutic agent that can curtail pro-tumoral inflammation and synergize with other therapies to enhance malignant cell apoptosis. Further clinical and translational evaluation of emavusertib as a single agent and in combination with other agents is merited.

## Author contributions

RP wrote the manuscript. MI, HT, CR, and RR assisted in research for the article and critical review and editing of the manuscript. All authors contributed to the article and approved the submitted version.
